# Identifying aspects of physiotherapy and occupational therapy provision in community palliative rehabilitation that could improve outcomes: A realist review

**DOI:** 10.1177/02692163251331166

**Published:** 2025-04-22

**Authors:** Jane Manson, Paul Taylor, Susan Mawson, Joanne Bayly, Carol Keen, Jacqui Gath, Tracy Green, Frances Anderson, Rob Smith, Alicia O’Cathain

**Affiliations:** 1 Sheffield Teaching Hospitals NHS Foundation Trust, Sheffield, UK; 2 Sheffield Centre for Health and Related Research, The University of Sheffield, Sheffield, UK; 3 The University of Sheffield, Sheffield, UK; 4 Cicely Saunders Institute, London, UK; 5 PPI Representative, Sheffield, UK; 6 St Luke’s Hospice, Sheffield, UK

**Keywords:** Palliative care, physiotherapy, occupational therapy, rehabilitation, models, community

## Abstract

**Background:**

The provision of physiotherapy and occupational therapy in palliative care is often poorly understood. There is currently no guidance on how to deliver these services in the community, potentially leading to unwarranted variation in practice and unmet patient need.

**Aim:**

To identify aspects of physiotherapy and occupational therapy provision in community palliative rehabilitation that could improve outcomes.

**Design:**

A realist review of the literature following RAMESES standards, with stakeholder input throughout.

**Data sources:**

Iterative literature searches were conducted from September 2023 to April 2024. All relevant data sources relating to delivery of physiotherapy and occupational therapy in community palliative care were included.

**Results:**

Forty-two international publications were included, published between 2000 and 2023. Five key aspects were identified: (1) Early referral into community palliative rehabilitation. (2) Layered model, basing level of service on complexity of needs. Within this, clinicians without professional qualifications deliver simple interventions after assessment by a qualified physiotherapist or occupational therapist while specialist clinicians review more complex presentations. Services are cohesive by being integrated with primary care, other community services and specialist medical and palliative care and there is representation of physiotherapists and occupational therapists within leadership teams. (3) Holistic assessments form the backbone of the service with personalised interventions tailored to patients’ needs and goals. (4) Accessible and flexible services are offered to meet patients’ needs throughout their palliative journey. (5) Information and education for patients and carers are available throughout.

**Conclusions:**

Integrating these five key aspects of physiotherapy and occupational therapy provision into community palliative rehabilitation could help ensure palliative patients receive the therapy they need.

**What is already known about the topic?**

- There is unmet need for community physiotherapy and occupational therapy for palliative patients.
- Physiotherapy and occupational therapy in the community can provide functional, psychological and social benefits to palliative patients, improving quality of life.
- Delivery of these rehabilitation disciplines in the community is inconsistent in palliative care despite the World Health Organisation stating that rehabilitation should be integrated within specialist palliative care.

**What this paper adds?**

- Provision of physiotherapy and occupational therapy in community palliative rehabilitation that follows a ‘layered’ approach, where non-specialists and specialists reviewing individuals according to need and complexity, could improve outcomes.
- Making community physiotherapy and occupational therapy available to patients at all stages of their palliative journey, tailored to patient need could improve outcomes.
- As well as being integrated with specialist palliative care, physiotherapy and occupational therapy in the community that is integrated with primary and other community services could improve outcomes.

**Implications for practice, theory or policy**

- If services are commissioned in the ‘layered’ model proposed it could help to ensure that patients are receiving the right service at the right time for their needs.
- Improved integration between physiotherapy and occupational therapy and specialist palliative care, and primary and community services, would improve communication about patient needs.
- Understanding the needs of patients and caregivers is likely to be key to delivering a holistic and flexible model of care. This could differ by health system to ensure the service meets the needs of the population.

## Background

Palliative rehabilitation aims to enable people with advanced progressive illness to live fully by optimising function and quality of life, while preventing and supporting decline until the end of life.^1,2^ It has grown as a speciality in recent years due to increased understanding of its utility, with examples of provision in different European countries.^3^ However, there is unmet need for palliative rehabilitation worldwide for palliative patients.^3–5^ Although rehabilitation, including palliative rehabilitation, is a multidisciplinary approach, the focus on addressing a person’s capacity and function^6^ means that physiotherapists and occupational therapists are the professions most often represented in rehabilitation teams.^7,8^ When palliative rehabilitation is provided in the community, physiotherapists and occupational therapists regularly assess and treat patients at home or other venues outside of inpatient hospital or hospice wards.

Previous systematic reviews in palliative rehabilitation have focussed on specific interventions such as gait re-education^9^ or a single disease process.^10^ There is no review of how to deliver physiotherapy and occupational therapy as a whole within community palliative rehabilitation, seeing it as a complex intervention. It is timely to review the evidence base in this area. A traditional systematic review of evidence of effectiveness would lack utility currently because due to the deteriorating nature of palliative patients, it is difficult to measure the benefits of interventions using randomised controlled trials.

Realist reviews are becoming an increasingly common methodology as clinicians and academics appreciate the complexity of healthcare.^11^ Realist review methodology is a theory driven approach that aims to unpick and interpret complexity to produce transferable findings about how an intervention works, why it works, for whom and to what extent.^12–14^ These are expressed in multiple context-mechanism-outcome chains and are further consolidated as programme theory.^15^ Although the standard approach is to explore ‘what works for whom in what circumstances’, some realist reviews focus on what aspects of care are important to improve outcomes.^16–18^ Therefore, undertaking a realist review of the delivery of physiotherapy and occupational therapy in community palliative rehabilitation could create insights to inform practice in this field.

### Aim

To identify aspects of physiotherapy and occupational therapy provision in community palliative rehabilitation that could improve outcomes based on the literature.

## Methods

This review was based on Pawson’s five stages of realist review^14^ and followed RAMESES (Realist and Meta-narrative Evidence Synthesis Evolving Standards) training materials and publication standards.^19^ The five stages were (1) development of programme theory, (2) search process, (3) selection and appraisal of the literature, (4) data extraction and organisation and (5) data analysis and synthesis. It is recommended that these five steps are undertaken with a Stakeholder Group to refine programme theory and understand causal theory from different perspectives.^20^

### Stakeholder group

An eight-person stakeholder group was purposively selected and recruited. The group consisted of two senior academics (with expertise in realist methodology and palliative rehabilitation), two clinicians (one occupational therapist and one physiotherapist), one oncology rehabilitation service manager, two patient and public representatives and the project lead (physiotherapist and doctoral research fellow). Stakeholder meetings (*n* = 3) occurred bi-monthly. The initial meeting was used to deliver training on realist review methodology and helped to develop the initial programme theory; subsequent meetings were used to refine the results (see Supplemental Material 1 for meeting content).

#### Step 1: Development of initial programme theory

**Figure 1.**
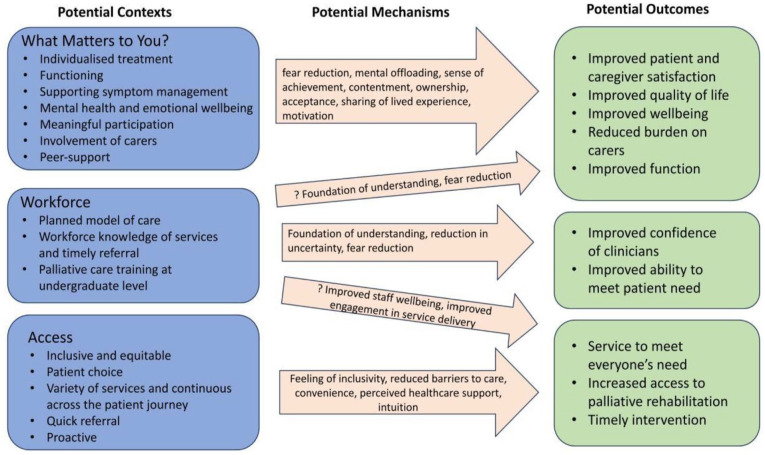
Initial programme theory based on initial literature search and stakeholder input.

When beginning a realist review, it is important to have a good understanding of the background literature and search strategy.^14^ An initial inductive approach was taken to familiarise the lead researcher with existing literature by identifying relevant references (*n* = 95) from two recent key general palliative rehabilitation guidelines.^2,3^ This helped to build initial programme theory and search terms. At the initial stakeholder meeting, participants were asked to brainstorm their views on what aspects of physiotherapy and occupational therapy in community palliative rehabilitation might improve outcomes from both a patient/caregiver and provider perspective. They were then asked to prioritise these thoughts to ensure the review was feasible in the given timeframe.^19^ Initial programme theory was then developed by the lead researcher based on this literature and stakeholder feedback (Figure 1).

#### Step 2: Search process

The search concepts were designed by the project lead and adapted based on feedback from members of the stakeholder group. The main concepts for searching were: palliative, physiotherapy and occupational therapy, community and quality of life (see Supplemental Material 2 for full search strategy). Due to the limited research in this area the terms were intentionally left broad to ensure that nothing was missed.

With the help of a research librarian, a search strategy was developed and piloted in one database (CINAHL) to ensure it produced relevant and manageable results. No changes were made, and six other databases were also searched (Cochrane Library, Web of Science, Global Health, SCOPUS, EMBASE and MEDLINE). Once the searches had been performed, it was identified that ‘hospice’ was missing from the initial search strategy. An additional search was completed in CINAHL including ‘hospice’, however this yielded no further articles therefore the researcher was confident that the original search terms were appropriate. Reference and citation searching was also completed for relevant literature. Initial searches took place on 3/11/23 and were repeated regularly by the librarian until 1/4/24 so that any new literature identified could be included.

In the second stakeholder meeting the initial programme theory was discussed alongside the initial search results. This comparison revealed gaps in the literature including: compassionate communities, support for caregivers and access. As these were important concepts for the stakeholder group, targeted searches were undertaken with these themes to see if there could be learning from the wider physiotherapy, occupational therapy or palliative care literature.

#### Step 3: Selection and appraisal of the literature

Search results were exported into Microsoft Excel.^21^ Duplicates and papers not meeting the inclusion criteria (Table 1) were removed. Screening was then undertaken in two phases by the lead researcher. Firstly, the results were screened by title and abstract. Secondly, the full texts of included documents were read and included based on rigour (the methodological strength of the article), and relevance (the significance it may have in contributing to the topic). About 20% of title/abstract searching was checked for consistency by another researcher (FA) and 20% full texts were discussed in one of the stakeholder meetings. Lack of rigour is not an absolute exclusion of literature in realist reviews^22^ therefore all literature which added to the programme theory were assessed independently using Joanne Briggs Institute checklists^23^ where appropriate. For transparency, a table of included studies can be found in Supplemental Material 4.

**Table 1.** Inclusion/exclusion criteria.

PICOS	Inclusion/exclusion
Participants/population	Inclusion: Adults with advanced, progressive illness^24^ receiving physiotherapy or occupational therapy or both in a community setting Exclusion: Participants under the age of 18. Participants who are undergoing curative treatment for their disease.
Intervention	Inclusion: Physiotherapy and occupational therapy palliative care delivered in the community – this can include intervention in patients’ own homes, outpatient facilities or community centres as well as day rehabilitation units. Exclusion: Pulmonary and cardiac rehabilitation programmes as these are already known to be effective models of care for patients with advanced, progressive cardiac or respiratory conditions. Interventions delivered in inpatient hospice or acute settings.
Comparator/control	Not applicable to this review although some literature may include controls and any control will be included.
Context	Inclusion: All data on how, why, when, for whom and to what extent aspects of adult community physiotherapy and occupational therapy work or do not work.
Outcomes	Any patient-centred outcome for example, but not limited to: improved functional, improved patient and caregiver satisfaction, more personalised care, more integrated care, better communication, better quality of life.

#### Step 4: Data extraction and organisation

All included publications were exported into NVivo for coding purposes. These publications were then ranked using a ‘red (most relevant), amber, green (least relevant)’ system and the most relevant read first. Articles were initially coded inductively where large conceptual buckets (themes) were created . Once red and amber texts were reviewed, green texts could be coded more deductively based on existing codes and initial programme theory. Coding was retroductive in nature meaning that if a mechanism was not fully explained in the text, a ‘best guess’ based on other literature or clinical experience was used.^25^

#### Step 5: Data analysis and synthesis

A narrative synthesis of the data was undertaken as follows. Sense was initially made of the literature using mind maps (see Supplemental Material 3 for an example) to understand what contexts, mechanisms and outcomes existed and crossovers between codes. The conceptual buckets were then refined, compared with the initial programme theory and placed into a framework where they were populated with data creating and supporting CMOC development.

Realist logic of analysis was used to draw conclusions from the extracted data.^14^ This included creating CMOCs (see Table 2) to inform and refine the programme theory^19^ and iteratively going back and forth between the data and CMOCs using realist conceptual tools of situating, juxtaposition, consolidation and reconciling to guide the process.^15^

**Table 2.** Conceptual buckets and CMOCs.

Conceptual bucket	Identifier	Context-mechanism-outcome chain
Early referral	ER1	When palliative rehabilitation teams engage early (C), relationships are formed between the patient and rehabilitation/palliative care services (M) which helps foster a feeling of comfort for the patient (O)
	ER2	When patients feel comfortable either in the rehabilitation environment or with the individual therapist (C), they are more likely to trust the therapist (M) leading to increased engagement (O)
	ER3	When relationships are formed between therapists and patients/caregivers (C), trust is formed between the therapist and patients/caregivers (M) which can lead to the patient/caregiver opening up more to the therapist (O) which in turn can lead to better identification of need (O)
Layered model within an integrated service Specialist and Generalist	LM1	When generalist therapists are able to provide community palliative physio/occupational therapy to non-complex patients (C), specialist therapists can provide a more timely response (O) because they are only called upon when needed (M)
	LM2	When patients who have complex needs are able to access specialist palliative rehabilitation (C), they are more likely to have their needs met (O) due to the specialist clinician being able to communicate more effectively and anticipate the patients’ needs (M) .
	LM3	Patients are more likely to have better physiotherapy and occupational therapy outcomes (O) when they are referred to specialist palliative care therapists (C) due to their increased understanding of treatment options and referral pathways (M)
	LM4	When there is joint working between specialist and generalist staff (C), there is improved care for patients (O) because the generalist staff are empowered and have the confidence to do more (M)
	LM5	However if there is no integration of specialist services leading to poor communication (C), patient experience may be poorer (O) due to unclear role boundaries (M)
Non-qualified staff and volunteers	LM6	When volunteers and generic assistants are able to administer prescribed rehabilitation interventions (C), there is a greater availability of more specialist staff (O) due to task delegation based on skill level (M)
	LM7	When volunteers and generic assistants are able to administer prescribed rehabilitation interventions (C), there is a cost-saving (M) meaning that resources are able to be spent on other areas of rehabilitation (O)
	LM8	When volunteers are engaged to provide compassionate communities (C), social connectedness is increased (M) which can support patients and their families to manage their symptoms and challenges at home (O)
Integration	LM9	When palliative physiotherapy and occupational therapy is integrated within specialist medical and palliative care teams (C), there may be earlier and more appropriate referrals (O) because there is a sharing of professional knowledge and roles (M)
	LM10	When clinicians have poor knowledge about community palliative rehabilitation and referral pathways (C), they have reduced awareness and confidence about when and where to refer (M) which could lead to later, inappropriate or missed referrals (O)
Leadership	LM11	In paternalistic palliative care services, having representation for rehabilitation in leadership teams (C) can lead to a culture of independence rather than ‘disablement through caring’ (O) because leaders empower clinicians to enable patients (M)
Holistic, patient-centred, individualised service	HO1	When patients have complex needs, providing a holistic assessment (C) can improve patient control (M) which can lead to greater satisfaction (O)
	HO2	When patients have complex needs, providing a holistic assessment (C) can help patients find focus and meaning (M) which can lead to meaningful engagement in activities (O)
	HO3	When patients have unrealistic expectations a, holistic, patient-centred assessment (C) can help patients reframe their own expectations (M) which can improve resilience and coping (O)
	HO4	When patients have limited time and energy, interventions that are tailored to their needs or interests (C) can improve motivation (M) which can improve self-management (O)
Offering patients a choice of flexible services to meet their needs	FL1	When palliative patients, who have an uncertain disease trajectory are offered a variety of rehabilitation models and settings (C), they should be able to engage with physiotherapy and occupational therapy at all stages of their illness if needed (O) as rehabilitation will always be accessible for them (M)
	FL2	When patients who are unable to leave their house are provided with physiotherapy and occupational therapy in their home (C), there is greater inclusion for patients (O) because service become more accessible for them (M)
	FL3	As palliative patients may have lots of hospital/hospice appointments, offering physiotherapy and occupational therapy sessions while they are already attending a hospital/hospice site (C) could improve attendance in rehabilitation (O) by reducing patient burden (M)
	FL4	As palliative patients already have a lot of hospital/hospice appointments, offering the option of education and self-management strategies (C), could improve engagement in rehabilitation (O) because the patient is not burdened by attending extra appointments (M)
	FL5	As palliative rehabilitation services are often resource-poor, offering group-based activities (C) might improve timely access for patients to palliative rehabilitation (O) because they are more cost- and time-effective (M)
	FL6	As palliative rehabilitation services are often resource-poor, offering hybrid and virtual models of rehabilitation (C) might improve timely access for patients to palliative physiotherapy and occupational therapy (O) because they reduce geographic obstacles (M)
	FL7	When patients are unable or unwilling to access technology, virtual or hybrid models of physiotherapy and occupational therapy (C) could lead to an inequity of services (O) due to reduced access (M)
	FL8	Where patients are engaged with virtual models, smart technology (C) can facilitate rapid, tailored rehabilitation (O) due to the sharing of real-time information (M).
	FL9	Where patients have a preference about attending group or 1:1 sessions, providing options (C) could improve patient engagement (O) by empowering patients to select the model that fits with their comfort levels, preferences and needs (M).
	FL10	Palliative patients and caregivers often experience social death. Offering group sessions (C) can create collective identity, shared understanding, connectedness and unity (M) which can lead to improved psychological wellbeing (O)
	FL11	Offering group sessions (C) can also lead to social regeneration and a sense of normalcy for patients (M) which can help them to re-shape what it means to live with an incurable disease (O)
	FL12	As patients with minimal education and low income are less likely to access palliative rehabilitation, offering patient-centred interventions which ‘hold the patients’ hands’ (C) are more likely to engage these patients (O) as they feel valued and supported (M)
Information and education for both patients and carers	PE1	When palliative patients who don’t need physiotherapy or occupational therapy initially are provided with accessible information or advice about their disease trajectory (C), there is an increased awareness of future symptom burden (M) which could reduce patient distress and improve resilience (O).
	PE2	When palliative patients are provided with accessible information about how to manage common symptoms (C), they are empowered to self-manage which could lead to improved patient control over their illness (O).
	PE3	When palliative patients are proactively provided with information and education about rehabilitative services and how to access them (C), they have a better understanding about the services available to them (M) which could lead to more timely access and acceptance of services (O)
	PE4	When palliative patients and caregivers are provided with information about how an intervention should work and feel (C), this increases patient and carer confidence in the intervention (M), which may lead to improved uptake (O)
	PE5	When carers are provided with information around what to expect and how to manage the patients’ symptoms (C), they can care for the patient better (O), due to having a greater understanding about how to help the patient
	PE6	When carers are provided with information around what to expect and how to manage the patients’ symptoms (C), they may have better quality of life as a carer (O) due to improved confidence in their caring abilities (M)

Cross-case comparison of literature in other specialities was completed to enhance understanding. Due to resource constraints, topics could not be searched in their own right, but when there was literature known to the stakeholder group members in that area it was explored. For example, one of the exclusion criteria for the core search was pulmonary rehabilitation; however when there was no literature found on caregiver support within the initial search, one of the stakeholder group members (JB) highlighted research on caregiver support undertaken within the breathlessness literature. This was then explored in the additional targeted searches.

Programme theory and CMOC development involved extracting data directly from the included literature, discussion with the stakeholder group (see Supplemental Material 1) as well as ‘hunch-driven logic’ which utilises creativity combined with common sense.^26^ Literature on substantive theories was then explored to see if they supported the results and could provide further understanding grounded in theory. See Figure 3 for intermediate programme theory with substantive theory.

## Results

### Publications found

**Figure 2.**
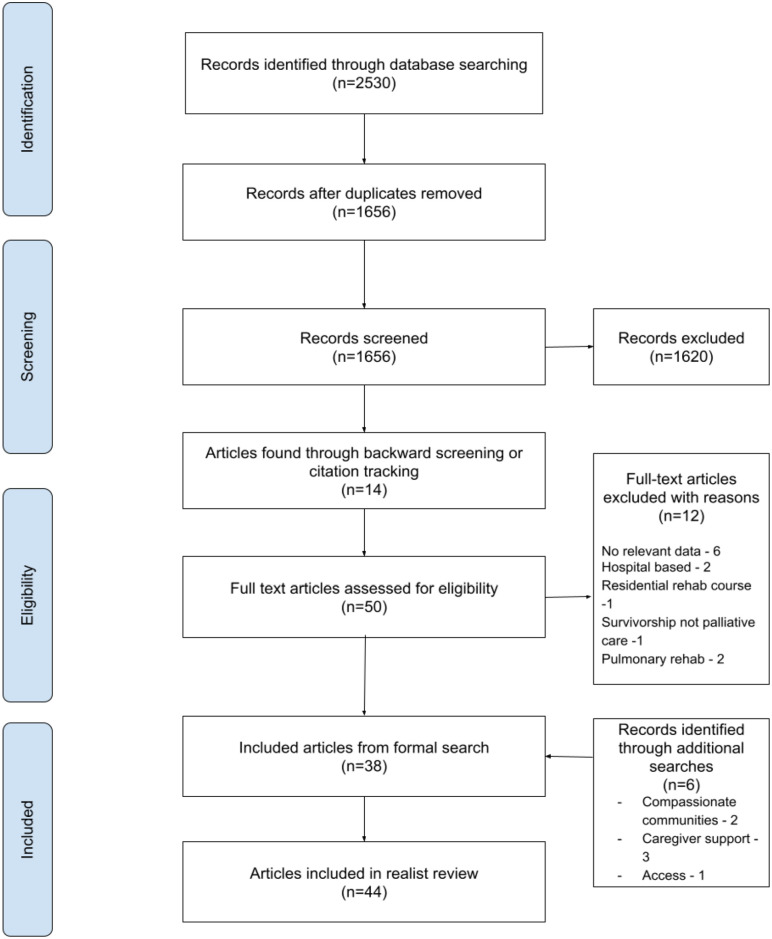
PRISMA diagram of included studies.

In total 2530 articles were identified through database searching with an additional 14 through citation searching and backwards screening. These were reduced to 50 for full-text screening due to duplication or relevance and 38 following the reading of the full-text. A further six articles were included from additional targeted searches (see Figure 2 for PRISMA diagram).

Forty-four articles were used to develop 36 CMOCs which were in turn used to refine the initial programme theory (see Table 2 for CMOCs). Documents dated from 2000 to 2023 and included studies from the United Kingdom,^27^ United States,^3^ Australia,^2^ Canada,^2^ Norway,^1^ Denmark,^5^ India,^2^ Sweden^1^ and Israel.^1^ Twenty-four included both physiotherapy and occupational therapy intervention, 11 were physiotherapy specific and 9 were occupational therapy specific. Documents consisted of interventional studies,^5^ review papers,^10^ qualitative studies,^11^ mixed methods studies,^2^ policy reports^3^ and other (e.g. PhD thesis).^13^

### Conceptual buckets

Five key conceptual buckets (themes) were developed from the CMOCs (see Table 2): early referral to community palliative rehabilitation; layered model of physiotherapy and occupational therapy within an integrated service; holistic personalised individualised service; offering an accessible, flexible service; and information and education for both patients and carers.

### Final programme theory

The narrative synthesis undertaken within each bucket informed the final programme theory for what aspects of physiotherapy and occupational therapy in community palliative rehabilitation might improve outcomes. This final programme theory consisted of five aspects of community palliative physiotherapy and occupational therapy that could improve outcomes:

#### Early Referral to community palliative rehabilitation

If the patient is referred early into the service, it gives the therapist an opportunity to form relationships with the patient and caregiver.^2,27^ Trust and a feeling of safety are fostered in these relationships leading to greater honesty about symptom burden, concerns and goals.^27^ Once a therapist gains this in-depth understanding of the needs of a patient then they can more accurately meet those needs, whether that be goal attainment, symptom management or psychological support.^2,27^ Early referral is represented as an outcome in the intermediate programme theory causal chains as it is anticipated that if all contexts are followed then early referral will naturally occur as part of this. However, it is also recognised that early referral could be a context which is why it has been included in the final programme theory.

#### Layered model of physiotherapy and occupational therapy within an integrated service

Based on Cowie et al.’s^28^ model of rehabilitation following a cardiac event, a similar model is proposed here where patients can move between layers of service based on their complexity or need.

##### Specialist and generalist therapy

Generalist therapists (those who do not exclusively treat palliative patients) are important in community palliative rehabilitation, by allowing specialists to focus on more complex patients.^2,29^ Referral to specialist therapists in more complex situations ensures that the honed communication skills and knowledge of specialist treatments and pathways that specialist clinicians have are utilised where they will have the most benefit.^29–36^ It is also critical, however, that generalist and specialist therapy services are integrated so that expert knowledge can be passed on to more generalist practitioners thereby improving the quality of service they are providing.^31,35–37^ A greater understanding of the specialist’s role by generalist therapists could also result in more appropriate and timely referrals to specialist palliative rehabilitation.^36,37^

##### Support workers and volunteers

Support workers (clinicians such as therapy assistants who work within rehabilitation and do not have a physiotherapy or occupational therapy qualification) and volunteers are increasingly being used to deliver rehabilitation interventions with delegated authority.^2,34,38,39^ Using support workers in this way, allows generalist therapists and more specialist clinicians to utilise their time seeing more complex patients.^34,38^ It is important to highlight that patients value registered physiotherapy/occupational therapy intervention due to the specialist knowledge they impart. Therefore it may be important that a holistic assessment is carried out by a registered member of staff before delegating to support workers and volunteers.^30,40^

#### Integration

It is important for physiotherapy and occupational therapy in community palliative rehabilitation to be integrated within specialist medical and palliative care teams. This allows for a sharing of knowledge between therapists and medical professionals which can lead to earlier referrals between specialist services.^2,3,29,33,36,37,41–46^ It is also important that physiotherapy and occupational therapy is integrated within primary and other generic community services. It is likely that most referrals will come from these sources, therefore improved knowledge of what palliative rehabilitation services can offer will increase timing and quality of referrals.^2,3,29,37,41,42^

#### Rehabilitation representatives within leadership teams

Specialist palliative care services can often be ‘disabling through caring’ meaning that professionals and carers do things ‘for’ a patient through compassion rather than enabling them to do tasks themselves.^47^ If there is physiotherapy and/or occupational therapy representation in specialist palliative care leadership teams, then it may encourage a more enabling approach to care resulting in a less dependent patient cohort.^2,46^

#### Holistic, patient-centred, individualised service

Providing a person-centred, individualised service is reported as important. This is initiated through holistic assessment so that not only physical needs are understood but also functional, psychological and spiritual. Discussing patients’ needs holistically can empower them to address what matters to them, improving satisfaction and meaningful engagement with services.^2,30,40–42,47–49^ Focussing on what matters to the patient and helping them to reframe their goals relating to unmet physical, social, spiritual, psychological and functional needs could motivate them to self-manage and could also improve resilience and coping.^2,8,32,33,37,38,40,47,50–54^

#### Offering patients a choice of accessible, flexible services to meet their needs

Patients who have had a palliative diagnosis often have many medical appointments to attend as well as fluctuating symptoms such as fatigue, breathlessness, reduced mobility, difficulty sleeping and pain. This combination may make it difficult for a patient to attend regular outpatient or hospice-based therapy sessions. If a service can offer a variety of physiotherapy and occupational therapy models and settings, then access is improved to rehabilitation throughout their disease trajectory.^3,8,32,33,37,38,40,47,50–54^ Allowing for flexibility such as arranging outpatient appointments while a patient is already attending the hospital or hospice further reduces the burden on the patient.^32,33,40,46^

As well as location-based models, offering both group and 1:1 sessions can empower patients to choose a service which fits their preferences improving engagement and enjoyment in physiotherapy and occupational therapy.^8,32,33,46,47,54–57^ Group sessions, as well as being more cost- and time-effective for services, may provide social regeneration in this cohort of patients who often experience social death.^33,47,55,57^ Bringing together a group of patients with similar needs can foster a shared understanding and unity which might improve psychological wellbeing.^33,46,55,57–59^ On the other hand, there may be patients who do not want to, or cannot, engage with group-based interventions. It is still important that these patients have access to physiotherapy and occupational therapy on a 1:1 basis whether that is in an outpatient, home-based or virtual setting.^32,33,47,51,54,56,57^

Virtual or hybrid (interventions over the internet or telephone as well as face-to-face) delivery of physiotherapy and occupational therapy can benefit patients as geographical obstacles are reduced, leading to timely access to services.^3,42,51–53,60^. Barriers such as no access to equipment or the internet still exist in some populations therefore only offering these models might reduce access to community palliative therapy.^3,42,51^ Access to palliative physiotherapy and occupational therapy is also lower in populations with low income and education so these patients may engage better with services that actively guide a patient throughout their journey rather than a virtual option.^50,61,62^

While there is understanding in the rehabilitation literature that patients who have access needs, such as barriers to travel or an inability to leave their house, will require flexibility in services, there was a distinct gap around other access issues such as people from racialised and other marginalised communities such as LGBTQ+, people living with poverty, rural, homeless and people with dementia.

#### Information and education for both patients and carers

Information and education can be made available throughout the patient’s palliative journey. On diagnosis information should be available such as how to manage common symptoms such as breathlessness and fatigue, how to access community palliative physiotherapy and occupational therapy services and what these services can offer and knowledge about managing deterioration and what to expect when dying.^31,32,35,37,49,58^ If patients at the beginning of their palliative journey do not feel they need, or understand, the interventions that palliative rehabilitation can offer, offering this information early on can highlight when and who to call for help if the patient is deteriorating or needs support.^31,32,37,49,58^

If a patient does not want to attend palliative physiotherapy or occupational therapy, for example due to current treatment burden, fatigue or being unable to get to the setting where it is delivered, or if home-based rehabilitation is not available in a timely manner, providing information on how to manage common symptoms can empower patients to self-manage rather than feeling helpless about their decline.^32,35,58^

When the patient and caregiver are engaged in palliative rehabilitation services, information and education are also important. If the patient and caregiver are given advice and education about the underlying mechanisms of a specific intervention, they may be more likely to engage.^35,63–65^ This is particularly important when the symptom burden is high. Often patients and caregivers look for solutions to relieve their symptoms completely, which may be unrealistic. Therefore, information is important throughout their journey to reframe expectations alongside encouraging engagement.^2,42,57,66^

Finally, carer quality of life and experience can be improved with personalised education around what to expect and how to manage symptoms. When carers are doubly prepared in this way, they are prepared for how to manage the patient and their ultimate deterioration and have more confidence in doing so.^31,35,59,64^ This can also lead to cost-savings for health and social care.

## Contribution of substantive theory

**Figure 3.**
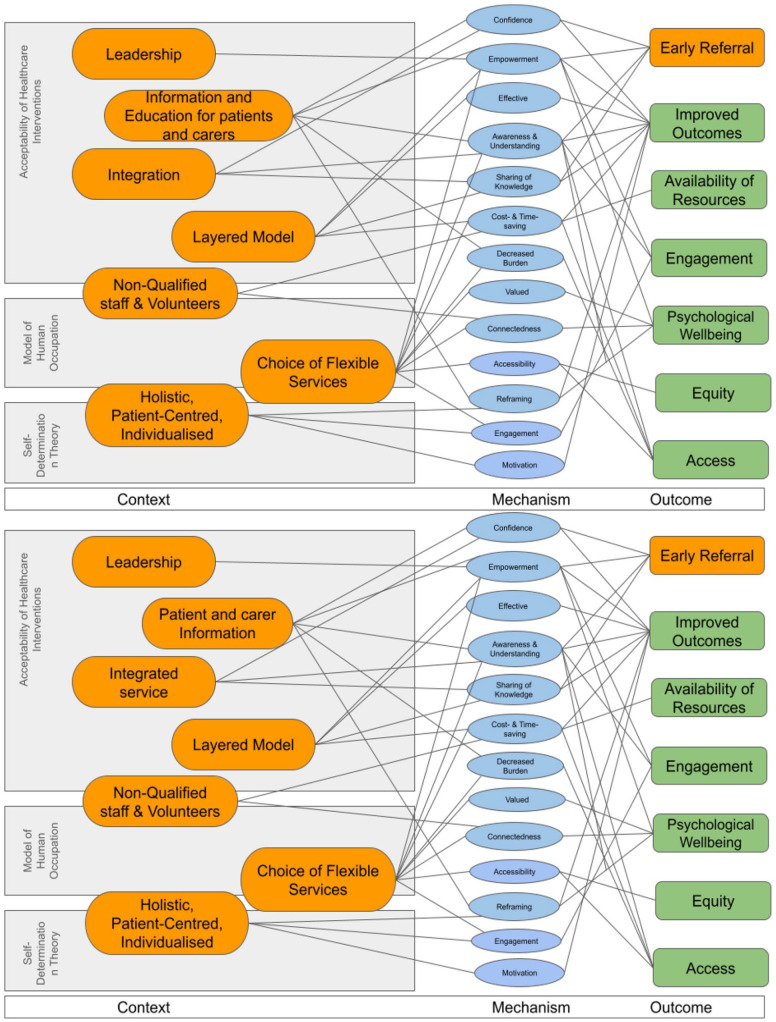
Iteration of programme theory with substantive theory. Orange colour indicates conceptual buckets.

There is a lack of substantive theories to support palliative physiotherapy and occupational therapy. However through contacting researchers in the field and searching theories related to the CMO chains above, the following substantive theories were found to support these conceptual buckets: the acceptability of healthcare interventions,^67^ the model of human occupation,^68^ and self-determination theory.^69^ These theories reflect upon patient motivation and volition and explain how extrinsic factors and intrinsic attitude can affect behaviour. A further explanation of each substantive theory is included below and an iteration of the programme theory is depicted in Figure 3 showing the overlaps with these substantive theories.

### Acceptability of healthcare interventions

A recent theory ‘acceptability of healthcare interventions’ argues that patients are more likely to adhere to treatment recommendations and therefore benefit from their outcomes if the intervention is considered acceptable.^67^ Patients can form judgements at different points during the intervention which can be categorised into ‘anticipated acceptability’ and ‘experienced acceptability’.^67^ Subcategories include affective attitude, burden, intervention coherence and self-efficacy.

Affective attitude and burden are likely to be connected to anticipated acceptability because if a patient does not understand community palliative physiotherapy and occupational therapy and its therapeutic approach, or perceives attending or participating to be a burden, then they will likely decline to participate, or not seek it out. This may occur when healthcare professionals do not fully understand community palliative physiotherapy and occupational therapy due to services not being appropriately integrated, so they do not explain these services correctly to patients. Patient burden could occur due to geographical barriers such as attending therapy at a hospital or hospice, or it could be related to performing an intervention that is not perceived to be centred around what matters to the patient - for example prescribing lower limb strengthening exercises when a patient’s main problem is low back pain. As highlighted in the final programme theory, it is important for therapy services to provide a holistic, flexible service so patient burden is reduced.

‘Experienced acceptability’ factors concern how an individual experiences an intervention. As explained in the section about information to patients and carers above, if an intervention is not adequately explained to participants, then disengagement may occur due to poor understanding of the intervention itself, or the mechanisms behind it. Finally, self-efficacy is a complex theory^70^ and is also related to good quality education at all stages of a patient’s journey, as the final programme theory indicate. If a patient feels confident to complete an intervention or self-manage, or if a caregiver is given adequate skills to be able to care for that individual, then they are more likely to be motivated to engage.

### Model of human occupation

A key model used by occupational therapists in practice worldwide, the model of human occupation, helps to recognise how and why activities are motivated, patterned and performed.^68^ This theory has been further defined within palliative rehabilitation in a literature review.^71^ The model itself contains three concepts which are said to influence the participation in meaningful activity: volition, habituation and performance. The main concept appropriate here is ‘volition’. This concerns how people value occupations and how they think and feel about their capability to participate and comprises a sense of capacity and self-efficacy.^72^ Volition is highlighted as being important at the end of life even if engagement is effortful due to the need to exert control during a transition to increasing dependency.^71^

Self-efficacy has been discussed above but Morgan et al explains that this could be taken further in palliative occupational therapy by the continual reframing of activities and prioritising during a patient’s decline.^71^ It highlights the importance of flexible, patient-centred models of community palliative rehabilitation described in the final programme theory so that patients can be supported to engage in occupations at all points throughout their palliative journey.

Sense of capacity is related to an individual’s perception and control over their physical, intellectual and social abilities.^73^ In relation to community palliative physiotherapy and occupational therapy, symptom burden, social isolation and the psychological impact of these can have a huge impact on an individual’s sense of capacity. As mentioned in the final programme theory above, offering group-based interventions can provide a sense of unity despite loss and could help to reverse social death. Offering flexible, holistic interventions can also give an individual more choice and therefore an increasing sense of capacity.

### Self-determination theory

Self-determination theory argues that individuals have evolved to be intrinsically motivated and this motivation is influenced by attitudes, values and behaviours.^69^ Within this theory there are three universal psychological needs: competence, autonomy and relatedness. It is therefore necessary that delivery of community palliative physiotherapy and occupational therapy is patient-centred and patients have the autonomy to make decisions about locations, delivery models and interventions while they are able to.

As mentioned previously in the model of human occupation, offering group-based interventions can bring individuals with similar symptoms together which can improve connectedness to other individuals, something which they might not get at home.

## Summary of final programme theory

**Figure 4.**
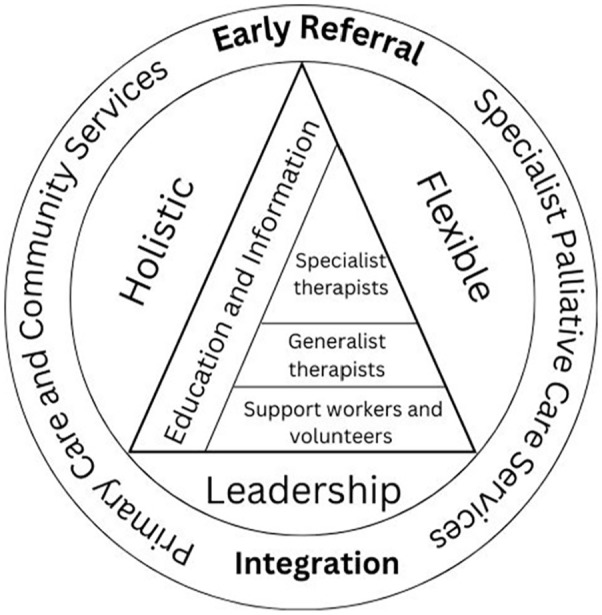
Summary of final programme theory: the aspects of physiotherapy and occupational therapy in community palliative rehabilitation that could improve outcomes.

Figure 4 (below) depicts a summary of the final programme theory of which aspects of physiotherapy and occupational therapy in community palliative rehabilitation the current literature suggests could improve outcomes. The layered model is at the centre with generalist therapists, support workers and volunteers delivering simple assessments and interventions, while specialist clinicians are available to review more complex presentations. There is a foundation of rehabilitation in leadership teams and holistic and flexible interventions form the backbone of the care delivered. Information and education is available for patients throughout their palliative journey. Early referral is key to the patient entering the service. Rehabilitation is integrated within primary care and community services as well as specialist palliative care.

## Discussion

This is the first review of any kind focussed on community palliative physiotherapy and occupational therapy. It consolidates international literature with stakeholder views to identify what aspects of physiotherapy and occupational therapy in community palliative rehabilitation could improve outcomes. This is supported by several substantive theories and aligns with international commissioning guidance for rehabilitation^74^ and integrating rehabilitation into palliative care.^3^ An alternative delivery model to the one presented here - a ‘one stop shop’ - has been proposed.^37,58^ Whereas this would improve integration between physiotherapy and occupational therapy and medical services, offering only this would reduce accessibility for palliative patients unable to leave their home.

Reflecting on the literature included in this review, it is notable that most of the studies were from the United Kingdom. At the end of the study, we reviewed the search strategy and reflected that it was inclusive of international literature. Articles not published in English were included in the search. It is possible that community palliative physiotherapy and occupational therapy is more developed in the United Kingdom than other countries, possibly due to the density of population making it easier to provide service close to home. Some supporting evidence for this is that 7 out of 20 European Association of Palliative Medicine palliative rehabilitation task force members are from the UK, followed by Denmark with 5.^75^ Denmark was the second highest publisher in this review

There were two important gaps in the literature. First, equality in access was important to the stakeholder group. While the literature identifies that the right model should be available to the palliative patient at the right time for them throughout their palliative journey^8,32,33,37,38,40,41,50–52,54,68,74^ there was a significant gap around how to improve this for individuals with health inequalities and other marginalised communities. Hospice UK is championing improving access for these populations^76^; however this has not yet been reflected in the rehabilitation literature. Second, despite campaigns urging people to talk about death and dying,^77,78^ the subject can remain taboo for many.^79^ Often rehabilitation professionals, support workers and volunteers spend significant time with patients and their caregivers. The trust built during this time can open opportunities to have conversations about symptom burden, patient and caregiver fears and planning for future deterioration which can improve quality of life for those individuals. However, those healthcare providers must feel confident to have these discussions and fear of having difficult conversations has been well documented in the palliative care literature.^80–82^ Despite being addressed in the stakeholder group and included in the initial programme theory, no literature was found on education for physiotherapists and occupational therapists in community palliative rehabilitation apart from direct supervision from more specialised colleagues.^31,35–37^ Wider literature indicates that undergraduate training for therapists in palliative care could improve self-rated knowledge and confidence in the field,^83,84^ however there is no evidence to suggest that this translates into clinical practice.

Information and education for patients and carers were identified as core to palliative care delivery^2,31,32,35,37,40,46,49,52,57–60^ and ere discussed in the literature in several forms: written, verbal, experiential and peer-support. Early in a palliative diagnosis patients and caregivers can be in shock^85^ and will not absorb information given. NICE^86^ highlights lack of information as one of the main inadequacies of palliative care delivery. It is therefore important to have a variety of information sources available so a patient or caregiver can access these in an appropriate form at an appropriate time.

### Strengths and limitations

This realist review followed RAMESES quality standards^19^ and has been reported transparently. CMOC development was guided through regular stakeholder discussion which included feedback from members of the public, clinicians and service leads ensuring that the outcomes are pragmatic and relevant to patients and services. The stakeholder group also contained two senior researchers: one experienced in realist research, and one specialising in palliative rehabilitation. This ensured the research was delivered and reported to a high standard. Inclusion of documents from around the globe as well as concordance of the CMOCs and programme theory with three substantive theories also improves the validity of the review.

There were four limitations. First, the authors identified the palliative care search filters developed by Rietjens et al.^87^ following reviewer feedback and considered whether these may have improved article identification. The authors then used Riejens’ search filters in CINAHL. One additional potentially useful article was found. However, on reading the full text it did not meet the inclusion criteria. The authors were therefore confident that the initial search terms were sufficiently robust. Second, the need to focus the review due to resource constraints.^19^ There was limited literature on caregiver and community support, access for marginalised populations, education for clinicians and virtual delivery of palliative physiotherapy and occupational therapy in the initial search. With further resources it might have been useful to undertake further searches in these areas within wider palliative care or rehabilitation literature. Third, many of the documents included were narrative reviews and qualitative evaluations. In the traditional hierarchy of evidence of effectiveness, these are seen as less reliable than randomised controlled trials.^88^ However, as the CMOCs were drawn from many documents, it allows these causal claims to be made.^89^ It is, however, important to interpret these results with caution until they have been empirically tested. Finally, although the inclusion of international research is identified as a strength, health systems around the globe run differently. The literature included in the review included all funding models (private and public) and through its inclusion adds further insight to the CMOCs and programme theory. Further investigation of the final programme theory and guideline production is planned, including eventual economic analysis but different funding models may inhibit implementation.

### Implications for practice and policy

Our final programme theory summarised in Figure 4 has potentially important implications for policy makers and commissioners of community palliative rehabilitation to help providers consider if they are delivering the best possible service to palliative patients and their caregivers. If services are commissioned based on the aspects proposed there would need to be better communication and integration not only between specialist and generalist colleagues, but also between registered clinicians and support-workers, the third sector, specialist palliative care and primary and community services. This may help to ensure that patients are receiving the right service at the right time for them. Understanding the needs of patients and caregivers is also key to delivering a holistic and flexible model. This may differ by population therefore care should be taken during the commissioning process to ensure the service is tailored to the needs of the population.

### Implications for research

The review has highlighted several gaps in the literature which would benefit from further investigation including:

- Access to physiotherapy and occupational therapy in community palliative rehabilitation by marginalised communities
- How to better support caregivers
- Education for generalist clinicians, including undergraduate education, and translation of this into practice.

## Conclusion

This is the first review of any type on the delivery of physiotherapy and occupational therapy in community palliative rehabilitation. The proposed final programme theory can be used to evaluate existing provision and guide future policy in this area. Gaps still exist including understanding access, support for caregivers in the community and education for generalist clinicians.

## Supplemental Material

sj-docx-1-pmj-10.1177_02692163251331166 – Supplemental material for Identifying aspects of physiotherapy and occupational therapy provision in community palliative rehabilitation that could improve outcomes: A realist review Supplemental material, sj-docx-1-pmj-10.1177_02692163251331166 for Identifying aspects of physiotherapy and occupational therapy provision in community palliative rehabilitation that could improve outcomes: A realist review by Jane Manson, Paul Taylor, Susan Mawson, Joanne Bayly, Carol Keen, Jacqui Gath, Tracy Green, Frances Anderson, Rob Smith and Alicia O’Cathain in Palliative Medicine
