# Supplementary material for: Identifying aspects of physiotherapy and occupational therapy provision in community palliative rehabilitation that could improve outcomes: A realist review
Source: Palliat Med. 2025 Apr 22;39(7):734–49. doi: 10.1177/02692163251331166 (PMC12227828; doi:10.1177/02692163251331166)
Supplement: sj-docx-1-pmj-10.1177_02692163251331166 – Supplemental material for Identifying aspects of physiotherapy and occupational therapy provision in community palliative rehabilitation that could improve outcomes: A realist review [file sj-docx-1-pmj-10.1177_02692163251331166.docx]

Supplementary Material 1: Stakeholder meeting dates and content

| Meeting Date & Time | Attendees | Content |
| --- | --- | --- |
| 6/12/2023 | 7 | Introduction to methodology  Initial programme theory discussion and development |
| 6/2/2024 | 5 | Search results and full-text discussion  Initial discussion about CMOCs |
| 13/5/2024 | 5 | Discussion around results and discussion |

Supplementary material 2: Search strategy

| **Element** | **Alternatives** | **Limiters** |
| --- | --- | --- |
| Palliative [Mesh] | “End of life” | Limiters -  Published Date: 19920101-202301103  ABSTRACT |
| Rehabilitation [Mesh] | physiotherap*  “physical therap*”  “Occupational therap*”  rehab* | Limiters -  Published Date: 19920101-202301103  ABSTRACT |
| Community | "Home-based"  "home based"  domiciliary  outpatient  "day centre"  virtual  hybrid  “tele-rehab*”  “tele rehab*” | Limiters -  Published Date: 19920101-202301103  ABSTRACT |
| “Quality of life” | "functional status"  pain  QoL  "mental health"  mood  acceptance  "advance* care plan*"  cost  integration  "hospital admission*"  "social care utilisation"  burden  "family support" | Limiters -  Published Date: 19920101-202301103  ABSTRACT |
| Boolean operators | ( Palliative.mp. OR "palliative care"[MeSH] OR "end of life".mp. ) | |
|  | AND | |
|  | ( (physiotherap*.mp.OR "physical therap*".mp. OR "Physical Therapy Modalities"[MeSH] "OR "occupational therap*".mp. OR "occupational therapy"[MeSH] OR rehab*.mp. OR rehabilitation [MeSH]) | |
|  | AND | |
|  | ("home-based".mp. OR "home based".mp. OR community.mp. OR domiciliary.mp. OR outpatient*.mp. OR outpatient[MeSH] OR "day centre".mp. OR virtual.mp. OR hybrid.mp. OR "tele-rehab*".mp. OR "tele rehab*".mp.) ) | |
|  | AND | |
|  | ( "functional status".mp. OR "functional status"[MeSH] OR pain.mp. OR pain [MeSH] OR "quality of life".mp. OR "quality of life" [MeSH] OR QoL OR "mental health".mp. OR "mental health" [MeSH] OR mood OR acceptance.mp. OR "advance* care plan*".mp. OR cost.mp. OR integration.mp. OR "hospital admission*".mp. OR "social care utilisation".mp. OR burden.mp. OR "family support".mp.) | |

Supplementary Material 3: Example of Mind Map


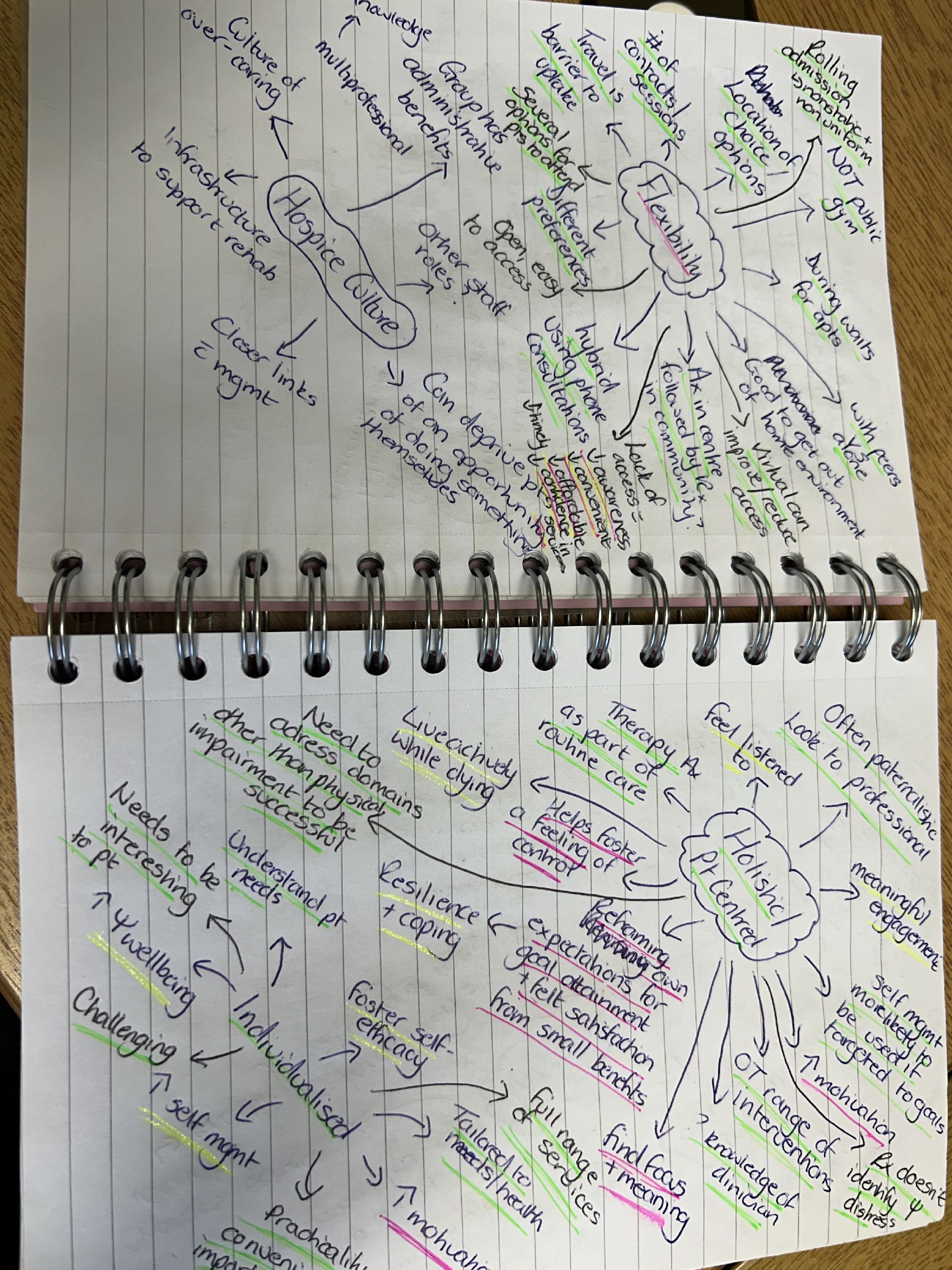


Supplementary Material 4: Table of included studies and study quality

| **Data Extraction Table, Study Quality and Contribution to CMOCs** | | | | | | | |  |  |
| --- | --- | --- | --- | --- | --- | --- | --- | --- | --- |
| **Author** | **Year** | **Country** | **Title** | **Study Design** | **Population Group** | **Setting** | **Objectives** | **Study Quality** | **Contribution to CMOCs** |
| Abel et al  (65) | 2018 | UK | Reducing emergency hospital admissions: a population health complex intervention of an enhanced model of primary care and compassionate communities | Cohort retrospective study | Primary care and compassionate communities | Primary care and community | To evaluate a population health complex intervention of an enhanced model of primary care and compassionate communities on population health improvement and reduction in emergency admissions to hospital | Moderate | LM8 |
| Badger et al  (66) | 2015 | UK | “It’s not about treatment, it’s how to improve your life”: The lived experience of occupational therapy in palliative care | Qualitative – Semi-structured interviews | Patients receiving occupational therapy | Inpatient and community | To investigate the lived experience of occupational therapy in palliative care from the perspective of patients. | High | LM2, LM3, HO1, HO2, HO4 |
| Bairapareddy et al  (67) | 2018 | India | Telerehabilitation for chronic obstructive pulmonary disease patients: An underrecognised management in tertiary care | Narrative review | Patients with COPD receiving telerehabilitation | Community | To enable the reader to compare the basics of telerehabilitation with other available rehab measures | Low | FL6 |
| Banks et al | 2022 | UK | The mountbatten rehab trifle recipe | Abstract for poster | N/A | Hospice | To present their rehabilitation approach at Mountbatten Hospice | N/A – abstract | LM6, LM7, FL1 |
| Bayly et al  (45) | 2018 | UK | Developing an integrated rehabilitation model for thoracic cancer services: views of patients, informal carers and clinicians | Qualitative – Focus Group | Patients affected by mesothelioma or lung cancer, their carers and clinicians providing their care | Community | To identify views of patients, carers and clinicians to develop and refine a rehabilitation model to be tested in a feasibility trial for people newly diagnosed with lung cancer or mesothelioma | High | HO1, HO4, FL1, FL2, FL3, FL4 |
| Bayly et al  (48) | 2022 | UK | Understanding the impact of the Covid-19 pandemic on delivery of rehabilitation in specialist palliative care services: An analysis of the CovPall-Rehab survey data | Cross-sectional national online survey | Rehabilitation leads for specialist palliative care services | Hospice, hospital and community | To understand rehabilitation provision in palliative care services during the Covid-19 pandemic | Moderate | FL1, FL6, FL7 |
| Bowker & Woods  (68) | 2017 | UK | Occupational therapist’s role to support patient goals with palliative rehabilitation | Poster abstract – reflective case study | One patient’s journey in the last weeks of life | Acute inpatient unit to home | To evaluate how an occupational therapist within an intermediate care service used palliative rehabilitative approaches to achieve the patient’s goals. | N/A – abstract | LM10 |
| Bradshaw et al  (69) | 2022 | UK | Group-based Tai Chi as therapy for alleviating experiences of social death in people with advanced, incurable disease: an ethnographic study | Ethnography | Patients with advanced, incurable disease | Hospice day therapy unit | To explore the personal and social experiences of participating in hospice-based Tai Chi among people with advanced, incurable disease, including its impact in mitigating experiences of social death | High | FL9, FL10, FL11 |
| Brighton et al  (58) | 2019 | UK | Holistic services for people with advanced disease and chronic breathlessness: a systematic review and meta-analysis | Systematic review and meta-analysis | Adults experiencing breathlessness related to advanced disease. | Outpatient or telephone | To examine the outcomes, experiences and therapeutic components of these services | High | PE2, PE4, PE5, PE6, LM3, LM4 |
| Burke et al  (70) | 2020 | UK | Physical activity in hospice care: A social ecological perspective to inform policy and practice | Qualitative - focus groups and semi-structured interviews | Patients and health providers from hospices | Hospice inpatient and outpatient | To explore perspectives on factors perceived important for influencing physical activity participation in hospice care | High | HO2, HO4, LM11, FL1 FL9, FL10, FL11 |
| Carson & McIlfatrick  (71) | 2013 | UK | More than physical function? Exploring physiotherapists’ experiences in delivering rehabilitation to patients requiring palliative care in the community setting | Qualitative - semi-structured interviews | Primary care physiotherapists | Primary Care | To explore the experiences of physiotherapists delivering rehabilitation in palliative care and to identify perceived barriers and enablers | Moderate | ER1, ER2, ER3, LM9, LM10, |
| Chahal & Chaudhuri  (49) | 2022 | India | Smart health rehabilitation in palliative care during the COVID-19 era | Narrative review | Patients with COVID-19 | Home, hospital or hospice | A review of smart health rehabilitation in palliative care | Low | FL1, FL6, FL8 |
| Cheville et al  (44) | 2017 | USA | Integrating function-directed treatments into palliative care | Narrative review | Patients with advanced cancer | Inpatient, outpatient, and community | To look at palliative care and rehabilitation through the prism of the advanced cancer population and doing the following: 1. Proposing a definition of “palliative rehabilitation”; 2. Reviewing palliative rehabilitation delivery care models; 3. Providing an overview of the barriers that sustain an underuse of rehabilitation services; and 4. Describing the strengths and weaknesses of our current rehabilitation interventions. | High | HO1, LM9, LM10, FL1, FL6 |
| Chow  (59) | 2020 | USA | How the utilisation of occupational therapy in end-of-life care is determined | PhD Thesis | Patients at the end of life receiving occupational therapy | Hospice and community | To investigate how the utilisation of occupational therapy in end-of-life care is determined | No tool available | LM3, LM4, LM5, LM9 |
| Chowdhury et al  (72) | 2020 | Australia | Cancer rehabilitation and palliative care – exploring the synergies | Narrative review | Patients with malignancy | All settings | Explore the under-recognised and underused parallels and synergies between the two specialties as well as identifying potential challenges and areas for future growth | High | HO1, HO3, HO4, LM9, LM10, FL6, FL7 |
| Farquhar et al  (73) | 2014 | UK | Is a specialist breathlessness service more effective and cost-effective for patients with advanced cancer and their carers than standard care? Findings of a mixed-method randomised controlled trial | Randomised controlled trial | Patients with advanced cancer | Home-based | To establish whether a breathlessness intervention service was more effective, and cost-effective, for patients with advanced cancer and their carers than standard care | Moderate | PE4 |
| Farquhar et al  (74) | 2017 | UK | Six key topics informal carers of patients with breathlessness in advanced disease want to learn about and why: MRC phase 1 study to inform an educational intervention | Qualitative – in-depth interviews | Patient-carer dyads living with breathlessness in advanced disease | All settings | To identify carers’ educational needs and explore differences by diagnostic group in order to inform an educational intervention for carers of patients with breathlessness in advanced disease | Moderate | PE4, PE5, PE6 |
| Hall et al  (75) | 2021 | UK | A randomised, feasibility trial of an exercise and nutrition-based rehabilitation programme (ENeRgy) in people with cancer | Randomised feasibility trial | Adults with incurable cancer | Home-based | To assess the feasibility of an exercise and nutritional rehabilitation programme in people with incurable cancer | Low | PE4, HO4 |
| Hopkins & Tookman  (76) | 2000 | UK | Rehabilitation and specialist palliative care | Narrative review | No specific population | All settings | To examine some of the recent developments affecting the provision of rehabilitative care to describe one specialist palliative care unit’s response to the challenge, highlighting the role of a nurse-led clinic within the service framework. | Moderate | LM9 |
| Hospice UK  (21) | 2021 | UK | Equality in hospice and end of life care: challenges and change | Report | Patients with a life-limiting condition | Hospice and community | To provide a snapshot for ourselves, and for the sector, of practice, academic research, progress and ways forward. | No tool available | FL13 |
| Kealey & McIntyre  (57) | 2005 | UK | An evaluation of the domiciliary occupational therapy service in palliative cancer care | Mixed Methods – structured interview | Patients in the palliative stage of cancer | Community | To evaluate the domiciliary occupational therapy service in a community trust for patients in the palliative stage of cancer from the patient and carer perspective | Moderate | HO4, LM2, LM4, PE3, PE6 |
| Lala & Kinsella  (77) | 2011 | Canada | A phenomenological enquiry into the embodied nature of occupation at the end of life | Qualitative – semi-structured interviews | Canadians 60 years of age or older who are diagnosed with a terminal illness. | Community | To examine the embodied nature of occupation at end of life from the perspectives of Canadians 60 years of age or older who are diagnosed with a terminal illness. | High | HO3, LM8 |
| Littlechild  (42) | 2015 | UK | Development of a hospice fatigue management programme – A review of group V individual sessions | Programme evaluation | Patients attending a fatigue management programme | Hospice outpatient | To provide patients with the opportunity to: learn how to manage the symptom of fatigue, enhance their self-confidence in the management of fatigue, and share their experiences . create support networks | N/A - abstract | HO4, FL1, FL9 |
| Loughran et al  (46) | 2019 | UK | Living with incurable cancer: what are the rehabilitation needs in a palliative setting? | Qualitative – semi-structured interviews | Patients living with incurable cancers | Hospital clinic and patient home | To address this paucity of information by recording and describing the lived experiences of people living with incurable cancer, the effects on their lives, their views on rehabilitation, and their perceived rehabilitation needs in palliative care setting. | High | PE1, PE2, PE3, LM2, LM10, FL1, FL3, FL4, FL9 |
| Maddocks et al  (78) | 2010 | UK | Exercise as a supportive therapy in incurable cancer: exploring patient preferences | Quantitative – survey | Patients with incurable cancer | All settings | To explore the acceptability of six programmes based on diﬀerent types of exercise to patients with incurable common cancers. | Moderate | HO4, FL9 |
| McEwen et al  (39) | 2016 | Canada | “I didn’t actually know there was such a thing as rehab”: survivor, family, and clinician perceptions of rehabilitation following treatment for head and neck cancer | Qualitative – focus groups | Survivors of head and neck cancer and their family members and healthcare professionals | All settings | To obtain first-hand contributions from survivors, family members, and front-line health care professionals regarding the rehabilitation needs for head and neck cancer (HNC) patients, to inform development of a rehabilitation intervention. | Low | PE3, LM4, LM5, LM9, LM10, FL1 |
| Mills & Payne  (79) | 2015 | UK | Enabling occupation at the end of life: A literature review | Narrative review | Occupation and occupational therapy at the end of life | All settings | To analyse and synthesise the literature to date, thus providing clarity and direction for future research and service provision. | High | PE3, HO1, HO2, LM10 |
| Nissen et al  (80) | 2022 | Denmark | Cancer rehabilitation and palliative care for socially vulnerable patients in Denmark an exploration of practices and conceptualisations | Narrative review | Socio-economically disadvantaged and socially vulnerable patients | All settings | To gather, synthesise and describe practice-orientated development studies presented in Danish-language publications and examine the underpinning conceptualisations of social inequality and vulnerability; explore related views of stakeholders working in the field. | High | HO4, FL12, FL13 |
| Nottelmann et al  (81) | 2021 | Denmark | Early, integrated palliative rehabilitation improves quality of life of patients with newly diagnosed advanced cancer. The Pal-Rehab randomised controlled trial | Randomised controlled trial | Adults diagnosed with advanced cancer within the last eight weeks | Outpatient and Community | We hypothesised that early integration of palliative rehabilitation could improve quality of life | High | LM9 |
| Nottelmann et al  (40) | 2019 | Denmark | A new model of early, integrated palliative care: palliative rehabilitation for newly diagnosed patients with non-resectable cancer | Narrative of development of a complex intervention | Patients with newly diagnosed advanced cancer | Outpatients and community | To describe a mode of palliative rehabilitation for newly diagnosed, advanced cancer patients and present data on how it was utilised during an RCT | No tool available | PE1, PE2, PE3, LM9, FL4, FL5, FL10 |
| Olesen et al  (82) | 2022 | Denmark | A cross-sectional evaluation of acceptability of an online palliative rehabilitation program for family caregivers of people with amyotrophic lateral sclerosis and cognitive and behavioural impairments | Qualitative - in-depth interviews and participant observations | Family caregivers of patients with ALS attending a new online palliative rehabilitation blended learning program | Virtual | Evaluate the acceptability of a new online palliative rehabilitation blended learning program (EMBRACE) for family caregivers of people with ALS and cognitive and/or behavioural impairments | Low | PE5, PE6, FL10 |
| Paltiel et al  (41) | 2009 | Norway | “The healthy me appears”: Palliative cancer patients’ experiences of participation in a physical group exercise program | Qualitative - semi-structured interviews | Palliative cancer patients with a life expectancy of 1 year who have completed a group exercise program | Outpatient cancer centre | To gain a comprehensive understanding of the patients’ perceptions and experiences of taking part in a group exercise program and to explore the meaning of such an intervention for the individual patient in order to provide valuable knowledge necessary for shaping future interventions in clinical practice | Moderate | LM2, LM3, LM9 FL1, FL3, FL9, FL10, FL11 |
| Phipps & Cooper  (83) | 2014 | UK | A service evaluation of a specialist community palliative care occupational therapy service | Mixed-methods - online survey and focus groups | Professionals who attend GSF meetings and who had made a referral to the specialist community occupational therapy service in the past 12 months | Primary care | To establish colleagues’ understanding of the palliative care occupational therapy service and its perceived benefit to patients in one local primary care area. | Poor | LM1, LM2, LM3, LM9, LM10 |
| Pilegaard et al  (84) | 2021 | Denmark | Rehabilitation and palliative care for socioeconomically disadvantaged patients with advanced cancer: a scoping review | Scoping review | Patients with advanced cancer who are socially disadvantaged | All settings | To map existing research of rehabilitation and palliative care for patients with advanced cancer who are socially disadvantaged | High | FL12, FL13 |
| Preston et al  (85) | 2023 | UK | Using volunteers to improve access to community rehabilitation in palliative care: the St Cristopher’s Living Well at Home Team | Service evaluation | Patients who were supported by a volunteer as part of the service improvement initiative | Community | To evaluate the service improvement project | No tool available | LM2, LM6, LM7, LM8 |
| Sviden  (86) | 2008 | Sweden | Therapeutic aspects of engagement in occupations in the context of day care for older people and patients in palliative care | PhD Thesis | Older people and palliative patients attending day care | Day centre and hospice | To identify and describe therapeutic aspects of engagement in occupations in day-care programs designed for older people with chronic conditions or patients in palliative care | No tool available | HO3, HO4, FL9, FL10, FL11 |
| Tennison et al  (87) | 2019 | USA | Frequency and characteristics of recommendations from interdisciplinary outpatient cancer rehabilitation monthly team meetings | Case series - descriptive study | Outpatient rehabilitation cancer patients discussed during consecutive monthly team meetings | Outpatients | To identify the frequency and characteristics of different types of recommendations that were derived through monthly interdisciplinary outpatient rehabilitation team meetings involving physiatrists, physical therapists, and occupational therapists | Moderate | LM9 |
| Tiberini & Richardson  (20) | 2015 | UK | Rehabilitative palliative care. Enabling people to live fully until they die. A challenge for the 21st century | Report | Patients with a palliative diagnosis | All settings | To describe rehabilitative palliative care, make the case for change, help hospices think about what this looks like in practice and provide guidance about how best to achieve it | No tool available | ER1, ER2, ER3, HO2, HO3, HO4, LM1, LM7, LM11, |
| Trend et al  (88) | 2002 | UK | Short-term effectiveness of intensive multidisciplinary rehabilitation for people with Parkinson’s disease and their carers | Quasi-experimental - before and after design | People with Parkinson’s disease and no cognitive impairment and their carers | Elderly care day unit | To evaluate the short-term effectiveness of an intensive multidisciplinary rehabilitation programme for people with Parkinson’s disease and their carers | Low | LM9 |
| Welford et al  (89) | 2023 | UK | Personalised assessment and rapid intervention in frail patients with lung cancer: The impact of an outpatient occupational therapy service | Service evaluation | Patients with thoracic malignancy and a clinical frailty scale score >5. | Lung cancer outpatient service | To evaluate the Lung Cancer Outpatient OT Service (LCOOTS) | No tool available | HO1, FL13 |
| Wheatley  (90) | 2015 | UK | The empowered living team: An innovative use of volunteers to provide rehabilitation and psychosocial support to hospice patients in the community | Conference abstract | Palliative patients in Newham, London | Community | To recruit and train volunteers to be able to visit patients at home to: Supervise rehabilitation/exercise programmes, provide complementary therapy, offer befriending/psychosocial support, practice self-management techniques, encourage participation in meaningful activities | N/A abstract | LM7 |
| World Health Organisation  (1) | 2023 | Denmark | Policy brief on integrating rehabilitation into palliative care services | Policy Brief | Palliative care services worldwide | All settings | To provide practical and actionable information and recommendations to support health ministers and leaders in health systems planning to integrate rehabilitation in palliative care services | No tool available | LM8, LM9, LM10, FL1, FL6, FL7 |
| Woldman  (50) | 2023 | Israel | Palliative physiotherapy in community settings for people with terminal illness | Retrospective chart review | Patients who received physiotherapy treatments during the last 6 months of their lives | Community | To explore the scope of palliative physiotherapy treatments that home dwelling people receive in Israel | Moderate | FL1, FL12 |
| Wosahlo & Maddocks  (43) | 2015 | UK | Benchmarking the provision of palliative rehabilitation within the hospice setting | Letter to the editor | UK hospices | Hospice inpatient, outpatient and community | To examine the range of AHP staffing, interventions, settings and evidence of service evaluation | Not enough information to appraise | FL5, FL1 |
